# Three-Dimensional Super-Resolution Morphology by Near-Field Assisted White-Light Interferometry

**DOI:** 10.1038/srep24703

**Published:** 2016-04-22

**Authors:** Feifei Wang, Lianqing Liu, Peng Yu, Zhu Liu, Haibo Yu, Yuechao Wang, Wen Jung Li

**Affiliations:** 1 State Key Laboratory of Robotics, Shenyang Institute of Automation, Chinese Academy of Sciences, Shenyang 110016, China; 2 University of Chinese Academy of Sciences, Beijing 100049, China; 3 Department of Mechanical and Biomedical Engineering, City University of Hong Kong, Kowloon Tong, Hong Kong

## Abstract

Recent developments in far-field fluorescent microscopy have enabled nanoscale imaging of biological entities by ingenious applications of fluorescent probes. For non-fluorescence applications, however, scanning probe microscopy still remains one of the most commonly used methods to “image” nanoscale features in all three dimensions, despite its limited throughput and invasiveness to scanned samples. Here, we propose a time-efficient three-dimensional super-resolution microscopy method: near-field assisted white light interferometry (NFWLI). This method takes advantage of topography acquisition using white-light interferometry and lateral near-field imaging via a microsphere superlens. The ability to discern structures in central processing units (CPUs) with minimum feature sizes of approximately 50 nm in the lateral dimensions and approximately 10 nm in the axial dimension within 25 s (40 times faster than atomic force microscopes) was demonstrated. We elaborate in this paper the principles of NFWLI and demonstrate its potential for becoming a practical method for high-speed and non-toxic three-dimensional nanoscale imaging.

Microscopy methods with nanoscale imaging capability in all three dimensions have been widely used in the life science, materials science, and industrial fields over the past two decades and have led to numerous advancements in applied research. Among these imaging methods, scanning probe microscopy (e.g., scanning tunneling microscopy and atomic force microscopy) has achieved molecular-level morphology resolution in three dimensions, but it is limited by its invasive and time-inefficient characteristics. Alternatively, optical microscopy provides a non-invasive, high-speed, and real-time imaging technique. However, its imaging resolution is limited to approximately half the illuminating wavelength (λ) in the lateral dimensions and several times deterioration in the axial dimension due to the loss of the evanescent waves induced diffraction barrier in far-field imaging^1^,^2^. During the last decade, the diffraction limit has been dramatically lowered with the advancement of super-resolution fluorescence microscopy methods, e.g., stimulated emission depletion (STED) microscopy^3^, structured illumination microscopy (SIM)^4^, stochastic optical reconstruction microscopy (STORM)^5^, and photoactivated localization microscopy (PALM)^6^. Fluorescence microscopes pave the way to identify specific molecules in three dimensions, however, the lost structural information restricts their potential applications in semiconductor wafer inspection or the exploration of many nanoscale materials and structures^7^,^8^. Near-field scanning optical microscopy (NSOM), which was developed by combining optical near-field interaction mechanisms and scanning probe methods, has been demonstrated to achieve optical super-resolution imaging in lateral dimensions without the assistance of optical contrast mechanisms, e.g., fluorescence^9^. However, the generation of topographical data via NSOM still relies on scanning probe methods; therefore, this technique suffers the same drawbacks as scanning probe microscopy in terms of time-inefficiency. One 3D morphology construction method fully based on optical principles and without the aforementioned problems is white-light interferometry (WLI), which has a vertical resolution (as high as 0.3 nm) comparable to that of scanning probe microscopy and is capable of surface profiling over a large area^10^. However, the lateral resolution of conventional WLI is restricted by the diffraction-limited optical resolution of the objective lens used.

Recently, efforts have been taken to surpass the lateral diffraction barrier in a more convenient manner; these efforts were pioneered by theoretical model results for a perfect slab lens with a negative refractive index for diffraction-free image reconstruction by the collection of evanescent waves^11^. Examples of relevant work include using 1) an optical superlens to restore sub-diffraction-limited images via the coupling of evanescent waves with the surface plasmon resonance of the silver film (60-nm half-pitch resolution at λ = 365 nm)^12^; 2) an anisotropic crystal with special dispersion and curvature to achieve subwavelength resolution^13^; 3) a cylindrical hyperlens to transform evanescent waves into propagating waves for sub-diffraction-limited imaging with a magnification factor (130-nm resolution at λ = 365 nm)^14^; 4) nanoscale lenses with plano-spherical convex structures resolving nanoscale features by near-field magnification (220-nm resolution at λ = 472 nm)^15^; and 5) optically transparent microspheres with a super-resolution focus for 50-nm-resolution imaging at λ = 600 nm (the main peak of the light source used)^1^. In general, imaging nanoscale objects using a microsphere superlens is a real-time, reliable, versatile, easy-to-use, and low-cost method compared to scanning electron microscopy (SEM) and has thus received considerable attention^1^,^16^,^17^,^18^,^19^. Subcellular structures (e.g., centrioles, mitochondria, and chromosomes)^18^ and 75-nm adenoviruses^19^ have been resolved using conventional optical microscopes assisted with microsphere superlenses. By introducing microspheres into the light path of a scanning laser confocal microscope, researchers have demonstrated 25 nm lateral resolution^19^. Enhancement of the resolution of endoscopes by microsphere superlenses has also been demonstrated^20^. However, this microsphere superlens technology has not been developed for 3D super-resolution imaging.

In this study, we report a new 3D super-resolution white-light microscopy method, near-field assisted white-light interferometry (NFWLI), that takes advantage of the topography profiling capability of WLI and the lateral sub-diffraction-limited resolution of microsphere-based superlenses. Spatially resolved features with a minimum size of approximately 50 nm in the lateral dimensions and ~10 nm in the vertical dimension are experimentally demonstrated without the use of fluorescent dyes. NFWLI is also shown to operate both in air and water, which increases its versatility and potential applications. Compared to scanning probe microscopy, this new technique offers substantial advantages for 3D nanoscale morphology profiling: much-improved time efficiency for large-area nanoscale morphology acquisition (i.e., field of view on the order of micrometers) and reduced repeatability errors (i.e., this technique does not require scanning probe tips that may wear out because repeated scans and cause surface profiling errors).

## Results

### Description of NFWLI

**Figure 1:**
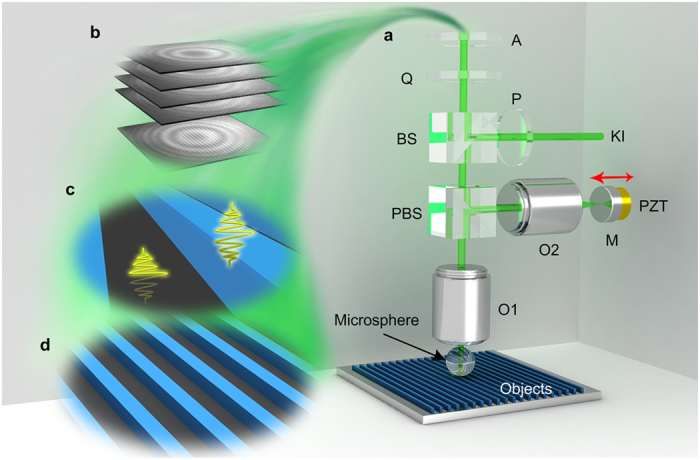
Near-field assisted white-light interferometry (NFWLI). (**a**) Schematic of the construction of the NFWLI system by integrating a microsphere superlens into a Linnik white-light interferometer. The components are as follows: analyzer (A), quarter-waveplate (Q), beam splitter (BS), polarizing beam splitter (PBS), polarizer (P), Köhler illumination (KI), mirror (M), piezoelectric ceramic scanner (PZT), and objectives (O1 and O2). (**b**) The frames of images are recorded during PZT linear scanning along the light path, as shown by the red arrow. (**c**) Analyses of the recorded interference images for 3D morphology construction. (**d**) Constructed 3D super-resolution morphology by NFWLI.

Figure 1 shows the basic concept of a NFWLI system. The system integrates a microsphere superlens with a WLI based on the Linnik configuration (Fig. 1a). The microsphere superlens magnifies objects and forms virtual images by collecting and transforming near-field information into far-field for super-resolution imaging^1^. Before experiments, these microsphere superlenses are randomly dispersed onto samples in air or in a water medium. The Linnik configuration was selected because it provides a convenient way to respectively adjust the object and reference arms (1) to compensate the optical path difference (OPD) induced by the introduction of microspheres into the object arm; (2) to focus the object objective (O1) onto the virtual image plane of the microspheres and regulate the reference mirror (M) position to refocus reference objective (O2) while keeping the two arms in balance. Objectives O1 and O2 are the same (Nikon TU Plan EPI ELWD, 50×, numerical aperture NA = 0.6). Because the objects are imaged in water, a coverslip with a thickness of approximately 170 μm was used to seal the BaTiO_3_ microspheres in water; another coverslip was placed between the objective O2 and mirror M to decrease the dispersion mismatch between the two arms. The use of coverslips effectively circumvents the influence of water evaporation and controls the water film thickness. The white-light source was incorporated into the NFWLI system by a Köhler illumination (KI) system, in which 3D movable and variable aperture and field stops are used to adjust the illumination conditions. As the difference of optical paths in both arms is within the coherence length (1.16 μm) of the white-light source, interference fringes can be generated (Fig. 1b)^10^. The field-of-view (FOV) of the NFWLI system is limited by the FOV of the microsphere superlens, which linearly increases with increasing sphere diameter^21^, and by the aperture and field stops in the Köhler illumination system. As the piezoelectric ceramic (PZT) scanner scans in the reference arm direction, a series of image frames containing interference fringes are recorded by a high-speed camera (Fig. 1b). After the analyses of the recorded image frames (Fig. 1c), the 3D super-resolution morphology is constructed (Fig. 1d).

### Experimental imaging performance of NFWLI

**Figure 2:**
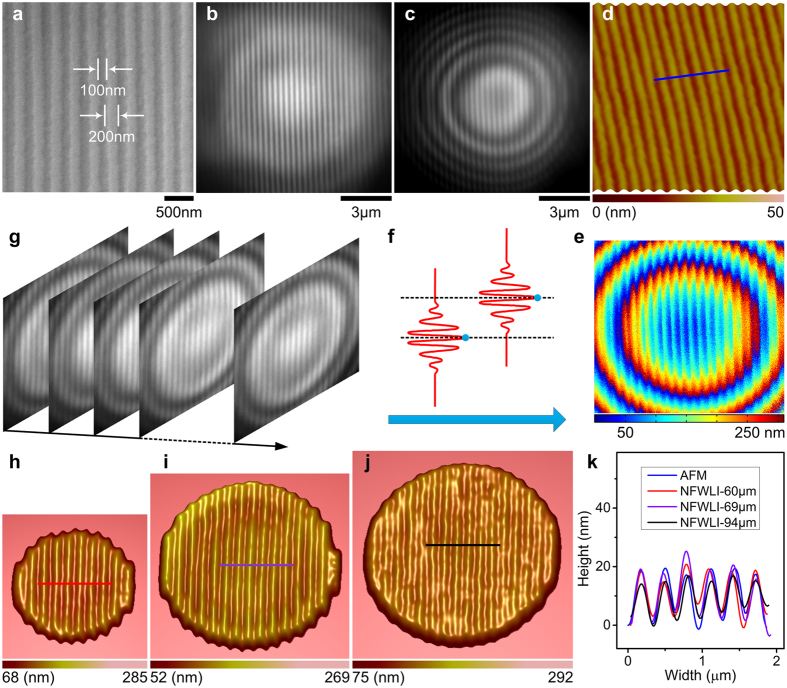
3D super-resolution morphology construction by NFWLI. (**a**) SEM image of structures on a Blu-ray disk surface after the transparent protection film was peeled off. (**b**) 2D super-resolution image by a microsphere superlens without NFWLI. (**c**) The interference fringes generated by NFWLI through the same microsphere superlens in (**b**). (**d**) 3D AFM scanning image of a Blu-ray disk surface. (**e**) Colored contour of the Blu-ray disk surface is constructed by searching the frames corresponding to the maximum intensity for each pixel (**f**) from the recorded interference fringes of a series of images (**g**). 3D morphology constructed by NFWLI through BaTiO_3_ microspheres with a diameter of (**h**) 60 μm, (**i**) 69 μm and (**j**) 94 μm. (**k**) Comparison of cross-sections marked by the lines shown in (**d**,**h**–**j**). These lines also represent the scale bars in each figure.

To demonstrate the discernment of feature sizes smaller than the diffraction limit in all three dimensions, we constructed the morphology of a Blu-ray disk’s surface by NFWLI through microsphere superlenses with different diameters, as shown in Fig. 2. The gratings on the Blu-ray disk surface consist of 200-nm-wide and 14–22-nm-high stripes spaced by 100-nm grooves; this surface was first imaged by SEM (Fig. 2a) and AFM (Fig. 2d). Figure 2b shows the virtual image generated by a 69-μm BaTiO_3_ microsphere in water; the scale bar is calibrated on the basis of SEM/AFM data. 3D contour of Blu-ray disk profile was obtained by programmatically searching the frame numbers corresponding to the maximum intensity for each pixel from the interference image series recorded by a high-speed camera. After calibration via the PZT step distance (∆z) related to two adjacent frames, we constructed 3D contours with real topographical dimensions (Fig. 2e). The frame rates used in the experiments shown in Fig. 2h–j are 103 fps, 100 fps and 293 fps, respectively, corresponding to ∆z values of approximately 0.37 nm, 0.38 nm and 0.13 nm, respectively. By using these contour data, we constructed 3D morphology images (Fig. 2h–j) after using low-pass filter and plane-fit processes. The AFM-scanned and the NFWLI-obtained profiles are consistent with each other (Fig. 2k). The local deviation of cross-section profiles shown in Fig. 2k originates from the non-uniform Blu-ray disk surface. The effective area of the generated 3D morphology image correlates with the FOV of the microsphere superlens, which increases with increasing diameter (Fig. 2h–j).

**Figure 3:**
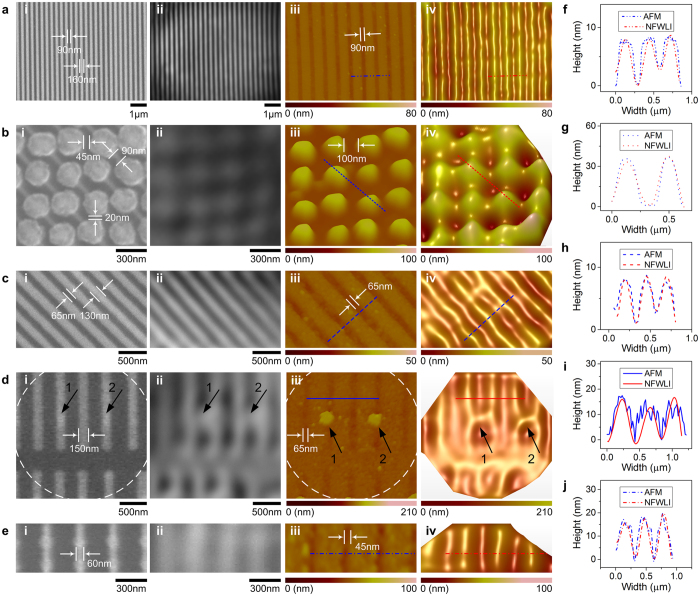
NFWLI used in semiconductor chip inspection. (**a**) 90-nm features, (**b**) nanodots array, (**c**,**d**) 65-nm structures and (**e**) 45-nm structures in a CPU chip. (i) In (**a**–**e**) are SEM images. (ii) In (**a**–**e**) are virtual images generated by a microsphere superlens. (iii) in (**a**–**e**) are AFM scanning images. The feature sizes shown in (iii) are full-width at half-maximum of the AFM scanned profile. (iv) In (**a**–**e**) are the 3D morphology constructed by NFWLI. (**f**–**j**) Show comparisons of the cross-sections marked by lines in (**a**(iii,iv)–**e**(iii,iv)), respectively. These lines in (**a**(iii,iv)–**e**(iii,iv)) also represent scale bars in each figure. The arrows shown in (**d**) correspond to the same positions where the circular features exist as shown in the AFM image. The possible reason why the circular features are difficult to resolve in the SEM image is that the material in the parallel vertical light-grey-color lines and the circular features are the same or the secondary electrons emitted from these areas are similar.

Figure 3 shows the sub-diffraction limit imaging of structures in CPUs by NFWLI through BaTiO_3_ microsphere superlenses with a diameter of 69 μm, 19 μm, 26 μm, 33 μm and 25 μm for the experimental results shown in Fig. 3a–e, respectively. The frame rate used in these experiments was 400 fps, corresponding to a ∆z of approximately 0.09 nm. The 3D morphology of nanoscale lines with a minimal feature size of ~50 nm (Fig. 3a,c,e) and complex shape structures (Fig. 3b,d) were clearly distinguished using NFWLI. The time required to record the interference images and data processing in NFWLI was approximately 25 s. The AFM images shown in this communication were obtained at a scan rate of 0.5 Hz with an image resolution of 512 × 512 pixels. The time used to acquire such an AFM image is approximately 17 min. Thus, the NFWLI also holds potential applications in semiconductor wafer inspection.

**Figure 4:**
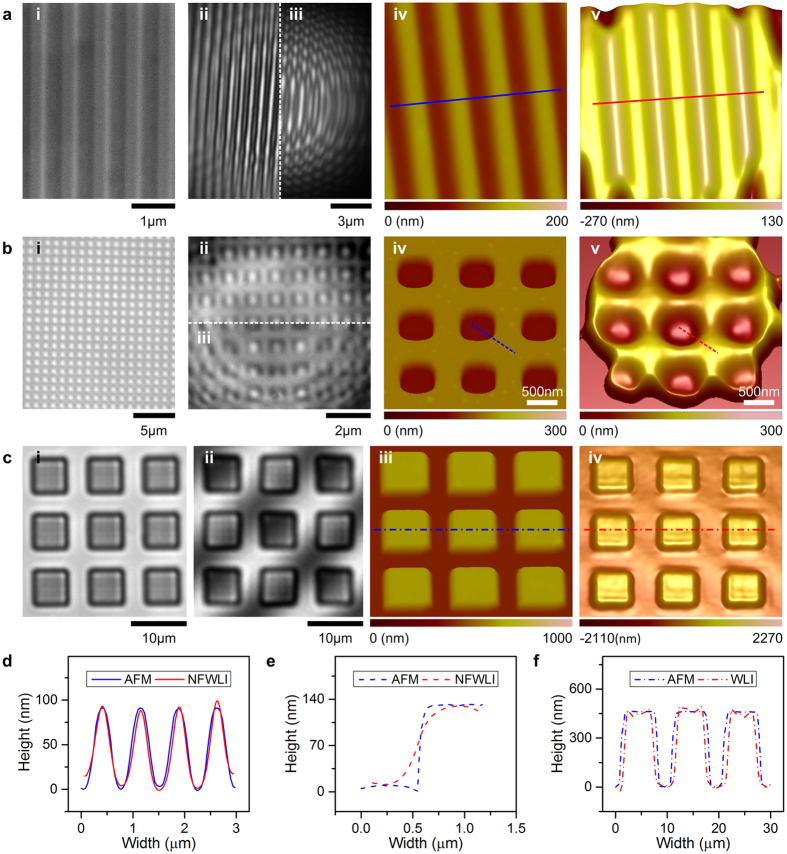
Verification of sub-diffraction-limited resolution of the NFWLI system in the vertical dimension. (i) Of (**a**) shows SEM image of structures on a DVD surface; (i) of (**b**,**c**) show optical microscope images (without assistance of microsphere superlens) of gridded structures. (ii) In (**a**,**b**) are virtual images generated by the microsphere superlens. (iii) In (**a**,**b**) are interference images generated by the NFWLI system through the same microsphere superlens in (ii). (ii) In (**c**) shows interference images of WLI. (iv) In (**a**,**b**) and (iii) in (**c**) are AFM-scanned images. (v) In (**a**,**b**) are 3D morphology constructed by the NFWLI system. (iv) In (**c**) is 3D morphology constructed by WLI. (**d**–**f**) Show comparisons of cross-sections marked by lines in (**a**(iv,v),**b**(iv,v),**c**(iii,iv)), respectively. The lines (in **a**(iv,v),**c**(iii,iv)) also represent scale bars in each figure.

To further investigate the acquisition capacity of NFWLI in the vertical dimension, a DVD disk’s surface (350-nm-wide and 90-nm-high stripes separated by 350 nm grooves) and a sub-microscale grid (500-nm-width and 120-nm-depth square pits spaced 500 nm apart) were imaged using a 65-μm-diameter BaTiO_3_ microsphere in water (Fig. 4a) and a 77-μm-diameter polystyrene microsphere in air (Fig. 4b), respectively. The frame rate used to record the interference images in Fig. 4a,b was 18 fps, and the ∆z was approximately 2.10 nm. The 3D morphology (Fig. 4a,b) construction by NFWLI follows the same process shown in Fig. 2e–g. A profile comparison between AFM and NFWLI measured data (Fig. 4d,e) confirmed the accuracy of NFWLI in the vertical dimension; NFWLI could be used to measure structures as high/deep as 120 nm. Surface profiling of a microscale grid (460-nm-high square with 10-μm pitch) by WLI was used as control experiments (Fig. 4c,f).

### Contrast enhancement by specific illumination and imaging mechanism analyses

**Figure 5:**
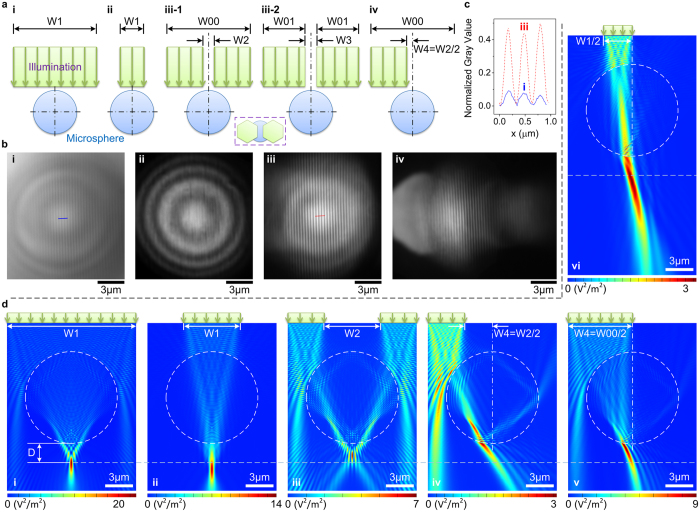
Illumination condition induced contrast enhancement. (**a**) Five different illumination conditions. The inset is the projection of conditions shown in (iii-1) or (iii-2). The hexagonal boundary is due to the shape of the irises used. (**b**) Comparison of the super-resolution imaging of the Blu-ray disc surface under different illumination conditions. These four results correspond to coaxial illumination covering a microsphere superlens (case (**i**) in (**a**) with W1 larger than microsphere diameter), coaxial illumination localized only in the middle area of the microsphere superlens (case (ii) in (**a**) with W1 smaller than the diameter), boundary illuminations (case (iii-1) in (**a**)) and partial illumination (case (iv) in (**a**)). The experimental result corresponding to case (iii-2) in (**a**) is similar to that in case (iii) in (**b**). The microsphere diameter is approximately 70 μm. For case (ii) in (**b**), the W1 is 48 μm. (**c**) Comparison of normalized gray values along the lines in (**b**(i)) and (**b**(iii)). The normalized gray values are calculated as (maximum − minimum)/minimum. (**d**) Simulated electric field intensity distribution under different illumination conditions.

Because the 3D super-resolution imaging by NFWLI is based on analyses of interference images, the quality of the sub-diffraction-limited features recorded by a camera influences the final constructed morphology. Previous research studies have experimentally demonstrated the use of polarized illumination to enhance image contrast^21^. Our studies demonstrate that image contrast can also be improved by specific illuminations. Figure 5a illustrates the five types of illumination conditions used in our studies: (i) coaxial illumination with adjustable area size (W1), which is larger than the microsphere diameter; (ii) coaxial illumination localized only in the middle area of the microsphere superlens, i.e., W1 is smaller than the microsphere diameter; (iii-1) boundary illumination with a fixed width (W00); (iii-2) boundary illumination with a fixed total illumination width (W01 = W00/2); and (iv) partial illumination of the case shown in (iii-1). These illumination conditions were experimentally realized by adjustment of the aperture and field stops in the Köhler illumination system. The paraxial images of the two stops in the microscope’s image space are schematically illustrated in the inset of Fig. 5a. Experimental results show that the image generated under the boundary illumination has the maximum contrast and that the virtual image of objects appears in the middle area of the microsphere when coaxial full-field or boundary illuminations are applied (Fig. 5b,c). The boundary illumination condition has been used to enhance the imaging properties of microsphere superlens when we conducted the NFWLI experiments (Figs 2, 3, 4). A comparison of the results in Fig. 5b reveals that the sub-diffraction-limited features were hardly resolved as the light did not transmit through the microsphere boundary (Fig. 5b(ii)) and the incident light through the middle area of the microsphere deteriorates image contrast (Fig. 5b(i,iii)). The origin of these phenomena related to the imaging mechanism of a microsphere superlens was further analyzed by calculating electric field intensity distribution around the microsphere under different illumination conditions based on finite-difference time-domain (FDTD) computational technique (Fig. 5d). The illumination conditions of the simulation results in Fig. 5d(i–iv) correspond to the experimental results in Fig. 5b(i–iv), respectively. As shown, a symmetrical focus is generated when symmetrical illumination is applied. The incident light transmitting through the middle area of the microsphere drives the focus away from the microsphere surface and broadens the focus (Fig. 5d). By contrast, illumination through the boundary area of the microsphere drives the focus to the microsphere surface and shortens the focus. Spectral analysis has demonstrated that, as the focus moves away from the microsphere surface, the effect of the evanescent field on shrinking the focus width is weakened because of its exponential decay property with increasing distance^22^. Thus, the evanescent field can be more easily coupled into the focus when the boundary illumination is exerted. However, this highly confined source field (focus) does not itself appear to be sufficiently strong to explain the super-resolution capability of a microsphere superlens because no confined focus is generated under partial boundary illumination (Fig. 5d(iv)), whereas sub-diffraction-limited details are resolved (Fig. 5b(iv)). Further comparison of the experimental results shown in Fig. 5b(ii,iv) and simulation results shown in Fig. 5d(ii,iv) indicates that the evanescent field generated from boundary illuminations is the main origin of the super-resolution capability of the microsphere superlens.

**Figure 6:**
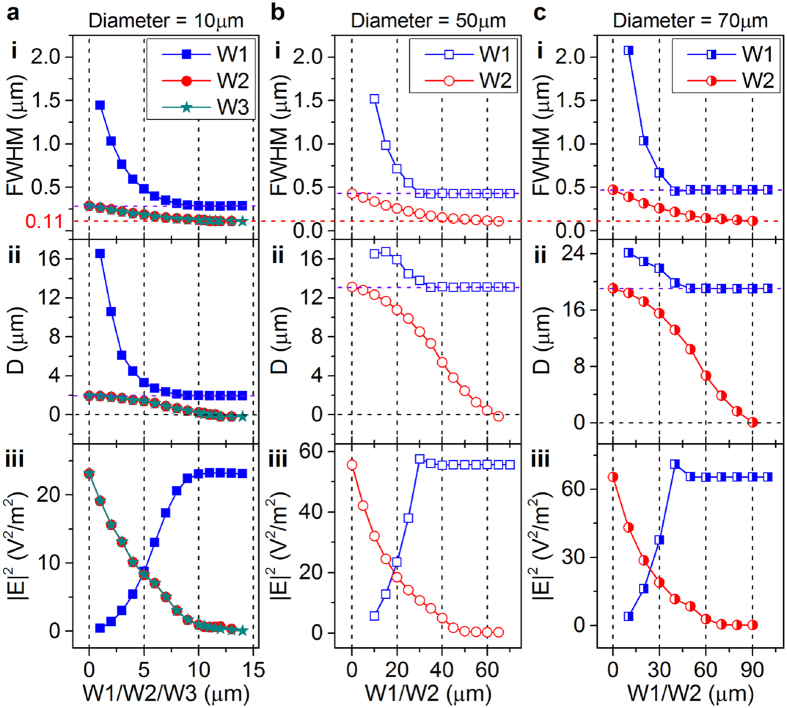
Illumination-condition- and microsphere-size-induced difference in the focusing properties. Simulated results show the relationship between the focus FWHM, position (D), the intensity of microspheres with diameters of (**a**) 10 μm, (**b**) 50 μm and (**c**) 70 μm, and the illumination size (W1/W2/W3). D represents the distance between the focus and microsphere apex, as shown in Fig. 5d(i). W1, W2 and W3 denote the illumination conditions shown in Fig. 5a(i,ii,iii-1, respectively, and are defined in Fig. 5a. W00 is 14 μm, 70 μm and 100 μm in (**a**–**c**), respectively. W01 is 7 μm in (**a**). The definition of the abscissa corresponds to the legend for the different curves.

Quantitatively simulated results describing how the focus properties, i.e., the full-width at half-maximum (FWHM), intensity (|**E**|^2^) and spatial positions (D), are influenced by different illumination conditions and diameters are shown in Fig. 6. The focus properties exhibit similar trends under the same illumination conditions as the microsphere diameter changes. Two boundary illumination conditions (Fig. 5a(iii-1) show the same focus properties (Fig. 6a), indicating that the illumination that does not transmit through the microsphere has negligible influence. As the light area (W1) increases, the focus FWHM decreases and the focus moves forward to the microsphere surface; however, the focus properties do not change when W1 approaches the microsphere diameter under coaxial illumination, especially at larger diameters (50 μm and 70 μm). Therefore, the contribution of the illumination near the microsphere boundary to the focus generation is negligible when middle illumination is present; i.e., the effects of evanescent waves generated from boundary illumination on focus are negligible compared with the waves caused by the light transmitting through the middle area. However, the boundary illumination can generate more tightly confined focus (approaching to approximately 110 nm) without obstruction from the middle illumination (Fig. 6), which we attributed to the contribution of evanescent fields^23^. Furthermore, the diameter of the inscribed circle of the hexagonal stops images, which induces the localized middle illumination (Fig. 5b(ii)), is approximately 48 μm and is considered to represent the illumination spot size. When this illumination condition is applied on a microsphere with a diameter of approximately 70 μm, the generation of super-resolved images is difficult (Fig. 5b(ii)). As the boundary illumination is added, the sub-diffraction-limited details appear in the virtual image (Fig. 5b(i)); however, it did not induce apparent changes in the focusing properties (Fig. 6c). These observations further confirm that the evanescent waves generated from the boundary illumination are likely the dominant factor for super-resolution imaging. The evanescent waves with high spatial frequencies caused by the boundary illumination could shift the large spatial frequencies of the sample into the propagating region^23^. The deletion of middle illumination decreases the scatter induced by propagation waves and weakens the obstruction on the propagation waves generated from large spatial frequencies of the sample. Therefore, the contrast is enhanced when only boundary illumination is applied. As the boundary illumination area shrinks, the focus intensity also decreases; however, this effect can be compensated by the increase of incident light intensity. These tightly confined focuses generated by boundary illumination also have potential applications in sub-diffraction-limited lithography^24^,^25^.

## Discussion

We have experimentally demonstrated that NFWLI could resolve features with sizes of approximately 50 nm in the lateral dimensions and ~10 nm in the vertical dimension. However, 10 nm is not the resolution limit for NFWLI because the resolution limit of WLI in the vertical dimension reaches the sub-nanometer level^10^. In addition, 25-nm lateral resolution has been reported by using microsphere superlenses^19^; hence, much room exists for improvement of NFWLI in all three dimensions. The tip effects of scanning probe microscopy, such as the broadening effect, inherently confine its applications for imaging structures with a large aspect ratio, which is not the case for NFWLI. Imaging of 90-nm-high and 120-nm-deep structures reported in this paper further verified the accuracy of NFWLI in vertical profiling data acquisition. The NFWLI system also provides a time-efficient method for 3D super-resolution imaging. For example, to image an 3.5 μm × 3.5 μm area while resolving sub-diffraction-limit features (e.g., as the images shown in Figs 1 and 3a), the NFWLI system will require approximately 25 s, whereas an AFM system requires approximately 17 min. In addition, this NFWLI image construction time could be further decreased through the development of more efficient algorithms and through the use of a higher-speed camera and scanner. To achieve a larger FOV, one can select a larger microsphere superlens under the condition that the resolution of the microsphere can satisfy the application requirement^21^, which is an easy approach. For the cases that the FOV or the resolution of bigger microspheres can not satisfy the actual requirement, one can develop a method to scan a microsphere superlens on the sample and then stitch the resulting images to form a large FOV image.

The successful construction of 3D super-resolution morphology by NFWLI could be partially attributed to the selected Linnik WLI system that provides a flexible configuration for adjusting the two respective arms to keep the optical path balanced as the microsphere superlens is introduced and the microscope objective is refocused on the virtual image plane of the microsphere superlens. Generally, evanescent waves oscillate as a function of time and along transverse directions^23^,^26^. They contain phase information and have the capability of interfering^27^. However, the vertical information of the sample cannot be directly constructed through analyzing the images generated by the interference of evanescent waves due to the lack of phase information along vertical direction. Nevertheless, in NFWLI the microsphere superlens converts the evanescence waves into propagating waves^1^,^21^,^28^. and constructs the phase information along the vertical direction from the evanescent waves. The converted propagating light containing phase information interferes with the reference light after transmission through the analyzer, as shown in Fig. 1a. Thus, the interference occurs in the far-field, which is similar to the experiments of Carniglia and Mandel^26^ and Cortes *et al*.^29^, i.e., the evanescent waves exist in the light beam but do not directly participate in the interference. But the original information of the sample was completely perturbed in the experiments of Cortes *et al*., which does not occur in NFWLI. For simplicity, the microsphere (and thin water film for BaTiO_3_ microspheres) was only inserted into the object arm, which introduces dispersion arising from the difference of geometrical path lengths in glass (and in water or microspheres) for the two arms. However, the dispersion error is not a concern because the dispersion deviation induced by the microsphere or water does not change in the FOV^30^. Achievement of high-quality interference images by properly regulating illumination conditions is another important factor for the realization of NFWLI.

In conclusion, we demonstrated that NFWLI can achieve super-resolution (i.e., below sub-diffraction limit) imaging in all three dimensions and has the potential advantage of imaging nanoscale structures much faster than conventional scanning probe microscopy methods. The accuracy and resolution in the vertical dimension is guaranteed by the WLI mechanisms and has been verified experimentally. In the lateral dimensions, the sub-diffraction-limited details are resolved in air and water by the assistance of near-field imaging using a microsphere superlens and imaging contrast is enhanced by application of specific white-light illumination conditions. This NFWLI method has potential applications in fields where fluorescence technology cannot be utilized and where detection of nanoscale structures with a large aspect ratio is needed, i.e., in cases where the tip of a scanning probe microscope cannot reach.

## Methods

### NFWLI system and imaging equipment

The BaTiO_3_ microspheres (supplied by Cospheric) or polystyrene microspheres (obtained from Alfa Aesar) were randomly placed on the sample surface. BaTiO_3_ microspheres were used in liquid medium (deionized water), and polystyrene microspheres were used for imaging in air. The optical components of a Köhler illumination system, analyzer, quarter-waveplate, beam splitter, polarizing beam splitter, polarizer and mirror used to assemble the NFWLI system were obtained from Thorlabs. The NFWLI system was fitted with a pair of 50× objectives (NA = 0.6, Nikon Plan EPI ELWD) and a high-speed scientific complementary metal oxide semiconductor (sCMOS) camera (PCO, Edge 5.5). The interferometric light path shown in Fig. 1 was coupled with the high-speed camera by a body tube (12× UltraZoom, 1-50503AD, Navitar). The mirror in the reference arm was driven by a piezo translation stage (NPX25-105, nPoint). The NFWLI system was illuminated by an intensity-controllable light source (C-HGFI, Nikon), and the middle wavelength was confined to approximately 550 nm by the optical components used. The 3D super-resolution images generated by the NFWLI system were constructed by AFM 3D software (NanoScope Analysis, Bruker). The low-pass filter and plane-fit processes were also conducted using this software. AFM-scanned results were obtained on a Dimension 3100 (Veeco, Inc). SEM images were collected using a Zeiss EVO MA10.

### Simulation method

Simulation results shown in Figs 5 and 6 were computed by Lumerical FDTD Solutions. These simulations were conducted at a light wavelength of 550 nm and under perfectly matched layer boundary conditions. The refractive indices of the BaTiO_3_ microsphere and water were set as 1.90 and 1.33, respectively. As shown in Fig. 6, the focus properties exhibit similar trends for different diameters under the same illumination conditions. The electric field intensity distributions around microspheres are also similar for different diameter microspheres. However, the simulated details of intensity distribution for smaller microspheres (10 μm) are clearer than those for 70-μm-diameter microspheres. Thus, Fig. 5d shows the intensity distribution of 10-μm-diameter microspheres.

## Additional Information

**How to cite this article**: Wang, F. *et al*. Three-Dimensional Super-Resolution Morphology by Near-Field Assisted White-Light Interferometry. *Sci. Rep.* **6**, 24703; doi: 10.1038/srep24703 (2016).
